# Cleaner synthesis of preclinically validated vaccine adjuvants

**DOI:** 10.3389/fchem.2023.1252996

**Published:** 2023-11-02

**Authors:** Alessio Romerio, Francesco Peri

**Affiliations:** Department of Biotechnology and Biosciences, Università degli Studi di Milano-Bicocca, Milano, Italy

**Keywords:** optimization, green chemistry, TLR4, glycolipid, medicinal chemistry

## Abstract

We developed synthetic glycophospholipids based on a glucosamine core (FP compounds) with potent and selective activity in stimulating Toll-Like Receptor 4 (TLR4) as agonists. These compounds have activity and toxicity profiles similar to the clinically approved adjuvant monophosphoryl lipid A (MPLA), included in several vaccine formulations, and are now in the preclinical phase of development as vaccine adjuvants in collaboration with Croda International PLC. FP compound synthesis is shorter and less expensive than MPLA preparation but presents challenges due to the use of toxic solvents and hazardous intermediates. In this paper we describe the optimization of FP compound synthesis. The use of regio- and chemoselective reactions allowed us to reduce the number of synthesis steps and improve process scalability, overall yield, safety, and Process Mass Intensity (PMI), thus paving the way to the industrial scale-up of the process.

## 1 Introduction

Vaccine introduction in 1798 rapidly decreased the morbidity and mortality of several deadly diseases, and their widespread use has been the reason for the eradication or attenuation of several pandemic diseases, including smallpox and the recent COVID-19 (Hilleman, 2000; Stewart and Devlin, 2006; Kayser and Ramzan, 2021; Zheng et al., 2022).

Many modern vaccines, the so-called subunit vaccines, only include parts of the pathogen, normally protein antigens, instead of the entire pathogen. They are therefore safer but less immunogenic than the vaccines containing the whole attenuated pathogen, thus requiring the addition of adjuvants (Delany et al., 2014).

Molecular adjuvants are chemical entities able to induce a strong, but controlled, immune response, thus increasing the efficacy of the vaccine in terms of the quality, intensity, and duration of immune response (Shah et al., 2017; O’Hagan et al., 2020).

The use of adjuvants contributes to the reduction of the amount of antigen required in a vaccine formulation. As normally the antigen is the most expensive component, adjuvants also have the potential to decrease the cost of vaccines, making them more accessible in developing countries (Delany et al., 2014; O’Hagan et al., 2020).

There is high industrial interest in this field: the market size of vaccine adjuvants has been valued at 895 million USD in 2021. This value is expected to double by 2027, with a forecast compound growth rate of 10.6% year-on-year, due to the involvement of companies such as GlaxoSmithKline PLC, Merck KGaA, and Croda International PLC (Industry Research, 2022).

However, the rate of innovation in the field of vaccine adjuvants has been extremely low in the last 20 years, and a formulation of aluminium salts (Alum) has been the only clinically approved adjuvant for years and even today very few compounds have been approved for human use (Li et al., 2008; Lambrecht et al., 2009; Shah et al., 2017).

**FIGURE 1:**
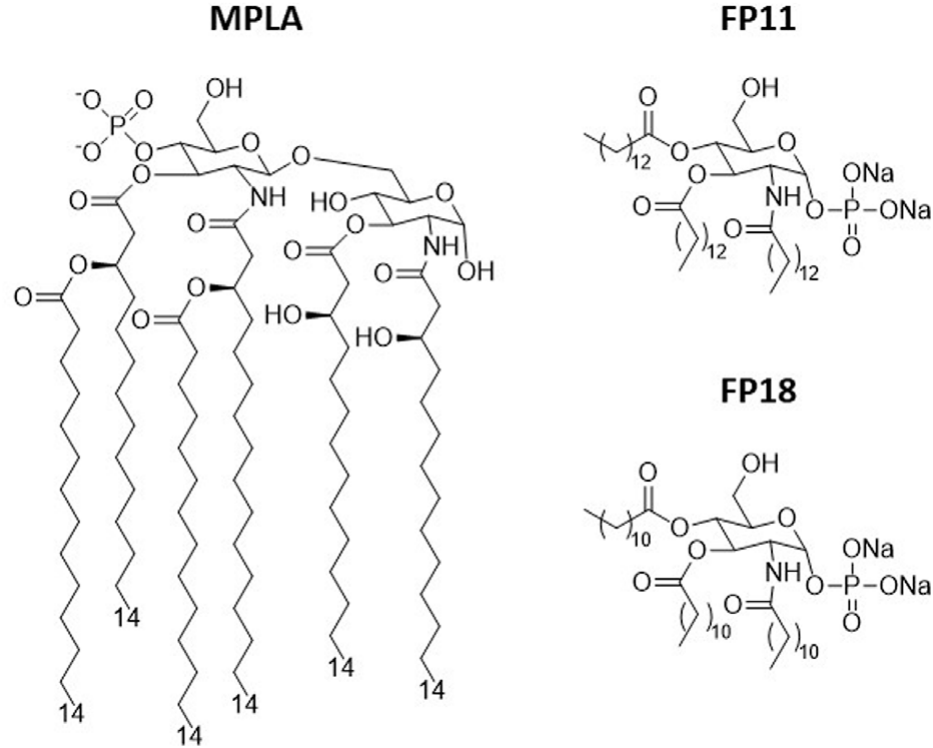
MPLA and FP compounds.

A clinically approved vaccine adjuvant is monophosphoryl Lipid A (MPLA, Figure 1), (Mata-Haro et al., 2007; Casella and Mitchell, 2008) a well-characterized Toll-like Receptor 4 (TLR4) agonist, included in several vaccine formulations (Cervarix^®^, Fendrix^®^, Shingrix^®^, Mosquirix^®^, Pollinex-Quattro^®^) (O’Hagan et al., 2020).

Synthetic MPLA (Avanti Lipids, United States) is a complex molecule, with a disaccharide core and linear and branched fatty acid chains with a stereogenic center at C-3, whose synthesis is long (>25 steps) with elevated production costs (the present cost of MPLA is ∼230 USD/mg in the American market). Furthermore, MPLA synthesis (699800P Avanti MPLA (PHAD^®^); Reed and Carter, 2014) is based on a massive use of “red” or environmentally undesirable solvents such as Pyridine or DMF (Alfonsi et al., 2008; Byrne et al., 2016; Joshi and Adhikari, 2019).

We recently synthesized **FP11** and **FP18** compounds (Figure 1), that showed to have similar activity to MPLA in inducing innate immune response in animal models of vaccination (Facchini et al., 2021). Their mechanism of action has been studied and it is based on the selective stimulation of the Toll-Like Receptor 4 (TLR4), one of the most important molecular switches of innate immunity (Peri and Minotti, 2019; Facchini et al., 2021). FP compounds possess however a simpler molecular formula and a shorter synthesis than MPLA. FPs retain most of the proinflammatory properties of MPLA when tested *in vitro* as well as the adjuvancy *in vivo* (Facchini et al., 2021). Due to their potential as cheaper substitutes for MPLA, we are developing FPs in collaboration with Croda International PLC as efficient substitutes for MPLA as vaccine adjuvants.

**SCHEME 1:**
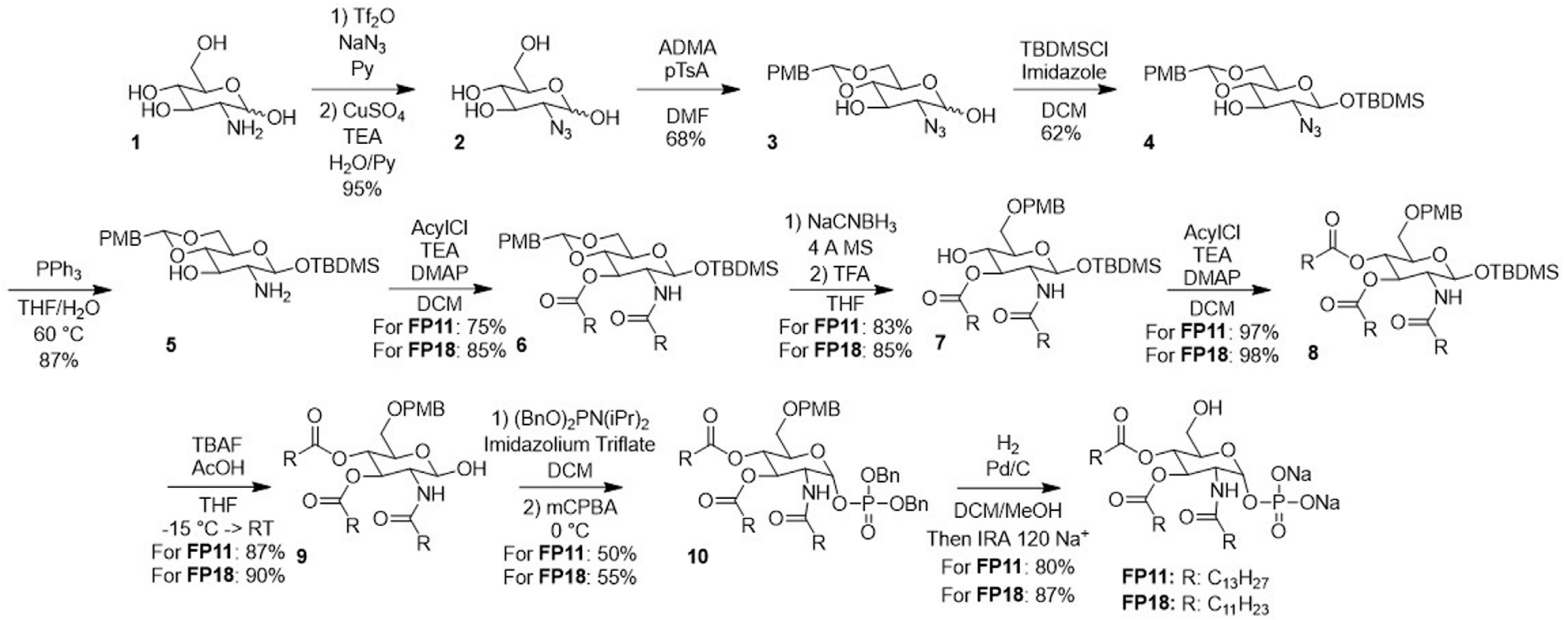
Previous synthesis of FP compounds.

Albeit definitively more convenient than MPLA, FP compound synthesis (Scheme 1) still presents some hurdles that may prevent industrial scalability and cause a significant environmental impact, having a Process Mass Intensity (PMI) of 3.0 × 10^4^ (Facchini et al., 2021). Furthermore, it employs a large amount of undesirable solvents (e.g., Pyridine, DMF, DCM), as defined by the Pfizer solvent bundle book, a widely accepted guideline for medicinal chemistry (Alfonsi et al., 2008; Byrne et al., 2016; Joshi and Adhikari, 2019). Here, we report a new, improved synthesis for FP designed with industrial scalability and environmental indications as guidelines. While the new synthesis still requires limited amounts of hazardous solvents, it is significantly shorter than the original one, translating in higher overall yields and lower PMI, a very important result due to the forecasted launch on the market of FP.

## 2 Results and discussion

We aimed to optimize the synthetic pathway of **FP11** and **FP18** (Scheme 1) in terms of the number of synthesis steps, safety, and environmental impact.

The published synthesis requires 10 steps with an overall yield of 7%. A chromatographic purification is required for 8 reaction steps out of 10, directly impacting the PMI of the process, calculated to 3.0 × 10^4^ (Peri and Minotti, 2019; Facchini et al., 2021).

Some synthetic steps have high safety and environmental hazards. For example, the first reaction requires the formation of a potentially explosive low molecular weight azide using the highly toxic pyridine as co-solvent; and high amounts of toxic and pollutant solvents such as DMF and DCM are abundantly used throughout the process.

Finally, the absence of chemical orthogonality between the protective groups does not allow for an easy selective deprotection, in the perspective of selectively functionalizing the C-6 hydroxyl group.

**SCHEME 2:**
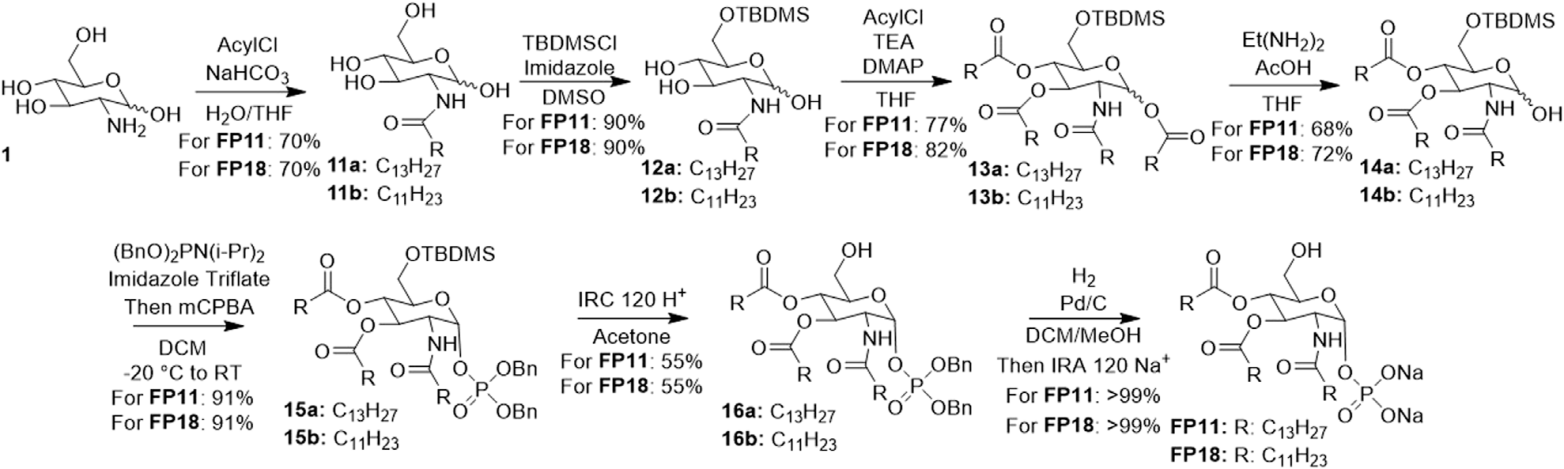
Cleaner synthesis for FP compounds, charcaterized by a reduced number of reactions, a limited use of red solvent, reduced PMI and increased yield.

A new, versatile synthesis has been designed (Scheme 2) with a reduced number of synthetic steps (7) and purifications and less toxic solvents involved. The overall yield is 18% and a PMI of 9.8 × 10^3^. This synthesis can be applied for both **FP11** and **FP18** by employing the correct lipid chain: reaction yields are very similar with a very narrow error range.

The first step is the acylation of the glucosamine on the 2-NH, exploiting its higher reactivity, so that it is not necessary to protect it anymore.

The second reaction is a silylation on the 6-OH of compound **11**, the only protection step in the synthesis: it is possible to regioselectively protect the more reactive primary alcohol over the other hydroxyls. However, the protecting group has to be carefully selected: a small one (e.g., TMS, TES) would not be selective enough; and a larger one (e.g., TBDPS, Trt) would prevent phosphorylation for sterical reasons. An additional challenge in this reaction was the choice of the solvent (Table 1), due to the poor solubility of the substrate both in aqueous and organic solvents. Initially, diluted pyridine was used, with a yield of 50%, but its extreme toxicity prompted us to search for a better medium. Several solvents were screened (i.e., MeCN, tBuOH, DMF) to no result, as the substrate failed to dissolve and the product was obtained only in traces. Finally, we managed to dissolve the substrate in DMSO at a low concentration (0.05 M) and to perform the reaction with a yield of 90%: therefore, we managed both to avoid pyridine and to reduce the PMI.

**TABLE 1:** Solvent screening for the silylation reaction.

Solvent	Concentration (M)	Imidazole (Eq.)	Temperature (°C)	Yield (%)
Py	0.1	-	70	50
Py	0,1	-	20	60
DMF	0.1	2.5	20	N/D
MeCN	0.1	2.5	20	N/D
DMSO	0.1	2.5	20	25
DMSO	0.05	2.5	65	20
tBuOH	0.05	1.5	85	N/D
DMSO	0.05	1.5	20	90

The third step of the pathway is the acylation of **12** on C-3 and C-4 hydroxyls. The reaction was first carried out in pyridine, which was eventually replaced with THF maintaining the high yield (80%) and reducing the hazards. The reaction stereochemistry at the anomeric carbon is dependent on the reaction conditions: short reaction time, high temperature and catalyst loading favor the formation of the thermodynamic α anomer, while longer reaction time, low temperature, and catalyst loading favor the formation of the kinetic β product (Romerio et al., 2023). As we have to remove the anomeric lipid chain (*v*. *infra*), the anomeric configuration is not relevant and it is possible to choose the protocol most suitable to one’s needs.

The fourth step is a regioselective deacylation of **13** with cleavage of the lipid chain in the anomeric position using a mixture of acetic acid and ethylenediamine. Interestingly, the anomeric acyl group acted as a leaving group in the presence of acids, and configuration seems to be retained (Zhang and Kováč, 1999).

Subsequent phosphorylation of **14** was performed using the phosphite to phosphate strategy, in which the compound undergoes a phosphitylation followed by one-pot oxidation to phosphate, similar to the previously published synthesis. This reaction is highly stereoselective: it always results in pure α configuration, independently from the starting configuration, as shown in several previous publications (Cighetti et al., 2014; Facchini et al., 2018; 2021; Peri and Minotti, 2019).

The 6-OH of **15** was then deprotected in mild conditions to avoid phosphate cleavage. Optimal cleavage conditions without concomitant reaction of protected phosphate consisted of the use of IRC 120 H^+^ resin in acetone. The reaction proceeded with 55% yield, but recycling of unreacted **15** allowed to further enhance yield.

Benzyl groups on the phosphates of compound **16** were removed by catalytic hydrogenation, as in the original synthesis (Peri and Minotti, 2019; Facchini et al., 2021).

The new synthesis is scalable for industrial production, with higher overall yield, lower PMI, and minimum use of “undesirable” or “red” solvents (Alfonsi et al., 2008; Byrne et al., 2016; Joshi and Adhikari, 2019).

## 3 Conclusion

Here we reported a new synthesis for FP compounds: a class of chemically simplified analogues of the known vaccine adjuvant MPLA, whose synthesis is significantly long and expensive. Regio- and chemoselective reactions allowed a drastic reduction in the use of protecting groups. Consequently, we managed to reduce the number of steps needed for the synthesis, which increased the overall yield (from 7% to 18%) and reduced the PMI (from 3.0 × 10^4^ to 9.8 × 10^3^) of the process. Furthermore, we eliminated the first hazardous intermediate and greatly decreased the use of red solvents replacing them with green or yellow solvents (Acetone, DMSO, or THF).

The described optimized synthesis will be further adapted to safety requirements and employed for industrial upscaling and production of the new immunostimulating agents **FP11** and **FP18**.

## 4 Materials and methods

All reagents and solvents were purchased from commercial sources and used without further purifications, unless stated otherwise. Reactions were monitored by thin-layer chromatography (TLC) performed over Silica Gel 60 F254 plates (Merck^®^). Flash chromatography purifications were performed on silica gel 60 60–75 μm from a commercial source. Solvent removal by rotavapor was carried out at 40 °C for most solvents and 55 °C for toluene and water, unless otherwise stated. ^1^H, ^13^C, and ^31^P NMR spectra were recorded with Bruker Advance 400 with TopSpin^®^ software, or with NMR Varian 400 with Vnmrj software. Chemical shifts are expressed in ppm with respect to Me_4_Si; coupling constants are expressed in Hz. The multiplicity in the ^13^C spectra was deducted by APT experiments. Exact masses were recorded with Agilent 6500 Series Q-TOF LC/MS System. The purity of the final compounds was about 95% as assessed by quantitative NMR analysis. Optical rotation values were acquired with Anton Paar MCP 100 polarimeter with a Type II cell (l = 100 mm; Ø = 5 mm) operating at 20 °C.

### 4.1 Compound 11a

*2-tetracanamido-2-deoxy-α,β-d-glucopyranose.*

Glucosamine hydrochloride **1** (10 g, 46 mmol, 1 eq.) and NaHCO_3_ (10.54 g, 124.2 mmol, 2.7 eq.) were dissolved in water (100 mL). Then, previously dissolved miristoyl chloride (12.5 g, 51.2 mmol, 1.1 eq.) in THF (100 mL) was added dropwise to the solution at 0 °C. A white solid started precipitating in the reaction flask. After 6 h stirring, the solution was filtered and a white solid was obtained, which was washed with 4 °C water. The solid was resuspended in 50 mL of HCl 0.5 M and stirred for 30 min. Afterward, the suspension was filtered again and the white solid was resuspended in 50 mL of THF. The white solid was again recovered by filtration. Excess water was then coevaporated with toluene under reduced pressure, to obtain the desired product **11a** as a white powder in 70% yield (12.50 g) as an anomeric mixture. The compound was used without further purification.

^1^H NMR (400 MHz, DMSO-d6) δ 7.64 (d, *J*_*NHβ*, *H-2β*_ *= 8*.*3 Hz*, 1H, NHβ), 7.50 (d, *J*_*NHα*, *H-2α*_ *= 8*.*0 Hz*, 4H, NHα), 6.46 (d, *J*_*1-OHβ*,__*H-1β*_ *= 6*.*3 Hz*, 1H, 1-OHβ), 6.41 – 6.36 (m, 3H, 1-OHα), 4.94 – 4.86 (m, 8H, H-1α+6-OHβ, 4-OHα), 4.78 (d, *J*_*4-OHβ*, *H-4β*_ *= 5*.*2 Hz*, 1H, 4-OHβ), 4.57 (d, *J*_*3-OHα*, *H-3α*_ *= 5*.*1 Hz*, 3H, 3-OHα), 4.53 (t, *J*_*3-OHβ*_, _*H-3β*_ *= 5*.*8 Hz*, 1H, 3-OHβ), 4.42 (dt, *J*_*6-OHα*, *H-6α*_ *= 11*.*5*, *J*_*H-1β*,__*1-OHβ*_ *5*.*5 Hz*, 5H, H-1β+6-OHα), 3.71 – 3.38 (m, 22H, H-3β, H-2α, H-2β, H-3α, H-4α, H-4β, H-5α, H-5β), 3.30 – 3.22 (m, 1H), 3.15 – 3.01 (m, 6H, H-6α, H-6β), 2.08 (dt, *J*_*CH2α*,_ _*CH2β*_ *= 10*.*9*, *7*.*4 Hz*, 10H, CH_2_α chains), 1.47 (q, *J*_*CH2β*, *CH2α*_ *= 7*.*0 Hz*, 11H, CH_2_β chains), 1.24 (s, 80H, chains bulk), 0.90 – 0.82 (m, 15H, CH_3_ chains).

^13^C NMR (101 MHz, DMSO-d6) δ 173.3, 172.8, 96.1, 91.1, 77.2, 74.8, 72.5, 71.6, 71.4, 70.9, 61.6, 57.6, 54.7, 40.6, 40.4, 40.2, 40.0, 39.8, 39.6, 39.4, 36.2, 35.8, 31.8, 29.5, 29.5, 29.4, 29.4, 29.2, 29.2, 29.1, 25.8, 22.6, 14.4.

HRMS (ESI-Q-TOF): m/z [M + Na^+^] calculated for C_20_H_39_NNaO_6_^+^: 412.2670. Found: 412.2674.

$$\alpha_{D}=+19.45$$

### 4.2 Compound 11b

*2-dodecanamido-2-deoxy-α,β-d-glucopyranose.*

Glucosamine hydrochloride **1** (5 g, 23.2 mmol, 1 eq.) and NaHCO_3_ (5.27 g, 63 mmol, 2.7 eq.) were dissolved in water (50 mL). Then, previously dissolved lauroyl chloride (5.60 g, 25.6 mmol, 1.1 eq.) in THF (50 mL) was added dropwise to the solution at 0 °C. A white solid started precipitating in the reaction flask. After 6 h stirring, the solution was filtered and a white solid was obtained, which was washed with 4 °C water. The solid was resuspended in 30 mL of HCl 0.5 M and stirred for 30 min. Afterwards, the suspension was filtered again and the white solid was resuspended in 30 mL of THF. The white solid was again recovered by filtration. Excess water was then coevaporated with toluene under reduced pressure, to obtain the desired product **11b** as a white powder in 70% yield (6.00 g) as an anomeric mixture. The compound was used without further purification.

^1^H NMR (400 MHz, DMSO-d6) δ 7.66 (d, *J*_*NHβ*, *H-2β*_ *= 8*.*0 Hz*, 1H, NHβ), 7.49 (d, *J*_*NHα*, *H-2α*_ *= 7*.*7 Hz*, 4H, NHα), 6.44 (d, *J*_*1-OHβ*,__*H-1β*_ *= 6*.*2 Hz*, 1H, 1-OHβ), 6.39 – 6.33 (m, 4H, 1-OHα), 4.95 – 4.91 (m, 5H, H-1α+6-OHβ), 4.89 (d, *J*_*4-OHα*,__*H-4α*_ *= 5*.*2 Hz*, 4H, 4-OHα), 4.79 (d, *J*_*4-OHβ*, *H-4β*_ *= 4*.*8 Hz*, 1H, 4-OHβ), 4.59 (d, *J*_*3-OHα*, *H-3α*_ *= 5*.*1 Hz*, 4H, 3-OHα), 4.51 (t, *J*_*3-OHβ*_, _*H-3β*_ *= 5*.*8 Hz*, 1H, 3-OHβ), 4.42 (dt, *J*_*6-OHα*, *H-6α*_ *= 11*.*5*, *J*_*H-1β*,__*1-OHβ*_ *5*.*5 Hz*, 5H, H-1β+6-OHα), 3.67 (dd, *J*_*H-3β*, *H-2β*_ *= 11*.*8*, *J*_*H-3β*,__*3-OHβ*_ *= 4*.*6 Hz*, 1H, H-3β), 3.61 – 3.40 (m, 14H, H-2α, H-2β, H-3α, H-4α, H-4β, H-5α, H-5β), 3.11 (ddd, *J*_*H-6αa*,_ _*H-6αb*_ *= 9*.*7*, *J*_*H-6αb*,_ _*H-6αa*_ *= 8*.*2*, *J*_*H-6αa*,__*H-5α*_ *= 5*.*1 Hz*, 4H, H-6α), 3.08 – 3.03 (m, 2H, H-6β), 2.08 (dt, *J*_*CH2α*,_ _*CH2β*_ *= 10*.*9*, *7*.*4 Hz*, 10H, CH_2_α chains), 1.47 (q, *J*_*CH2β*, *CH2α*_ *= 7*.*0 Hz*, 11H, CH_2_β chains), 1.24 (s, 80H, chains bulk), 0.90 – 0.82 (m, 15H, CH_3_ chains).

^13^C NMR (101 MHz, DMSO-d6) δ 173.3, 172.8, 96.1, 91.0, 77.2, 74.7, 72.4, 71.5, 71.3, 70.8, 61.5, 57.5, 54.7, 40.5, 40.3, 40.1, 39.9, 39.7, 39.5, 39.3, 36.1, 35.7, 31.7, 29.5, 29.5, 29.4, 29.4, 29.2, 29.2, 29.1, 25.8, 22.6, 14.4.

HRMS (ESI-Q-TOF): m/z [M + Na^+^] calculated for C_18_H_35_NNaO_6_^+^: 384.2361. Found: 384.2364.

$$\alpha_{D}=+47.63$$

### 4.3 Compound 12a

*2-tetradecanamido-2-deoxy-6-O-tert-butyldimethylsilyl-α,β-d-glucopyranose.*

To a solution of **11a** (3.0 g, 7.7 mmol, 1 eq.) and imidazole (785 mg, 11.5 mmol, 1.5 eq.) in dimethylsulfoxide (154 mL, 0.05 M) a solution of TBDMSCl (1.28 g, 8.5 mmol, 1.1 eq.) in DCM (13 mL) was added dropwise under an inert atmosphere in an ice bath. Subsequently, the solution was allowed to return to room temperature and stirred overnight. Reaction, monitored by TLC (DCM/MeOH 9:1; Rf product: 0.50), was then stopped and the solution was concentrated under reduced pressure. Then it was diluted with EtOAc and washed three times with NH_4_Cl. The organic phase thus obtained was dried with Na_2_SO_4_ and the solvent was removed by rotavapor. The crude product thus obtained (3.65 g) was resuspended in heptanes at 0 °C for 30 min. Then, the suspension was filtered under vacuum and the desired compound was recovered as a white solid. After filtration, 3.50 g of compound **12a** as a whiteish solid was obtained, in 90% yield.

^1^H NMR (400 MHz, MeOD) δ 5.12 (d, *J*_*H-1α*, *H-2*_ *= 4*.*2 Hz*, 0H; H-1α), 4.58 (d, *J*_*H-1β*, *H-2*_ *= 8*.*1 Hz*, 1H; H-1β), 4.03 – 3.92 (m, 1H; H-3), 3.83 (dd, *J*_*H-4*, *H-3*_ *=* 11.2, *J*_*H-4*, *H-5*_ *=* 5.3 Hz, 1H; H-4), 3.60 (t, J = 9.2 Hz, 1H; H-2), 3.49 – 3.22 (m, 5H; H-5, H-6), 2.25 (t, *J*_*CH2α*,_ _*CH2β*_ *=* Hz, 2H, CH_2_α chains), 1.64 (q, *J*_*CH2β*, *CH2α*_ *= 7*.*3 Hz*, 2H, CH_2_β chains), 1.33 (d, *J = 14*.*4 Hz*, 21H, Chains bulk), 1.03 – 0.80 (m, 14H; 3x CH_3_ chains + 9x tBu-Si), 0.21 – 0.04 (m, 7H; Me-Si).

^13^C NMR (101 MHz, MeOD) δ 175.9, 95.7, 76.8, 74.8, 70.7, 62.9, 57.3, 48.2, 48.0, 47.8, 47.6, 47.4, 47.2, 47.0, 36.1, 31.7, 29.4, 29.4, 29.2, 29.1, 29.1, 28.9, 25.6, 25.1, 24.8, 22.3, 17.9, 13.0, −6.5, −6.5.

HRMS (ESI-Q-TOF): m/z [M + Na^+^] calculated for C_26_H_53_NNaO_6_Si^+^: 526.3534. Found: 526.3542.

$$\alpha_{D}=-72.2$$

### 4.4 Compound 12b

*2-dodecanamido-2-deoxy-6-O-tert-butyldimethylsilyl-α,β-d-glucopyranose.*

To a solution of **11b** (3 g, 8.3 mmol, 1 eq.) and imidazole (850 mg, 12.4 mmol, 1.5 eq.) in dimethylsulfoxide (166 mL, 0.05 M) a solution of TBDMSCl (1.4 g, 9.1 mmol, 1.1 eq.) in DCM (14 mL) was added dropwise under an inert atmosphere in an ice bath. Subsequently, the solution was allowed to return to room temperature and stirred overnight. Reaction, monitored by TLC (DCM/MeOH 9:1; Rf product: 0.50), was then stopped and the solution was concentrated under reduced pressure. Then it was diluted with EtOAc and washed three times with NH_4_Cl. The organic phase thus obtained was dried with Na_2_SO_4_ and the solvent was removed by rotavapor. The crude product thus obtained (3.85 g) was resuspended in heptanes at 0 °C for 30 min. Then, the suspension was filtered under vacuum and the desired compound was recovered as a white solid. After filtration, 3.71 g of compound **12b** as a whiteish solid was obtained, in 90% yield.

^1^H NMR (400 MHz, DMSO-d6) δ 7.62 (d, *J*_*NH*, *H-2*_ *= 7*.*9 Hz*, 1H; NH), 6.43 (d, *J*_*1-OH*, *H-1*_ *= 6*.*4 Hz*, 1H; 1-OH), 4.90 (d, *J*_*4-OH*, *H-4*_ *= 6*.*5 Hz*, 1H; 4-OH), 4.77 (d, *J*_*3-OH*, *H-3*_ *= 9*.*1 Hz*, 1H; 3-OH), 4.42 (t, *J*_*H-1*, *H-2*_ *= 7*.*0 Hz*, 1H; H-1), 3.86 (d, *J*_*H-6a*, *H-6b*_ *= 10*.*8 Hz*, 1H; H-6a), 3.66 (dd, *J*_*H-6b*, *H-6a*_ *= 11*.*0*, *J*_*H-6b*, *H-5*_ *4*.*6 Hz*, 1H; H-6b), 3.30 (d, *J*_*H-2*, *NH*_ *= 7*.*9 Hz*, 2H; H-2 + H-3), 3.14 – 2.98 (m, 2H; H-4 + H-5), 2.06 (t, *J*_*CH2α*,_ _*CH2β*_ *= 7*.*4 Hz*, 2H CH_2_α chain), 1.48 (s, 2H; CH_2_β chains), 1.24 (s, 20H; chain bulk), 0.94 – 0.74 (m, 12H 3x CH_3_ chains + 9x tBu-Si), 0.05 (d, *J = 3*.*0 Hz*, 6H; Me-Si).

^13^C NMR (101 MHz, DMSO-d6) δ 173.2, 95.9, 77.1, 74.8, 70.8, 63.6, 57.5, 40.6, 40.4, 40.2, 40.0, 39.8, 39.6, 39.4, 36.2, 31.8, 29.5, 29.5, 29.4, 29.4, 29.2, 29.1, 26.4, 25.8, 22.6, 18.6, 14.4, −4.7, −4.7.

HRMS (ESI-Q-TOF): m/z [M + Na^+^] calculated for C_24_H_49_NNaO_6_Si^+^: 498.3226. Found: 498.3223.

$$\alpha_{D}=+50.4$$

### 4.5 Compound 13a

*1,3,4-tri-O-tetradecanoyl-2-tetradecanamido-2-deoxy-6-O-tert-butyldimethylsilyl-β-d-glucopyranose.*

Compound **12a** (1.0 g, 2.0 mmol, 1 eq.) was dissolved in anhydrous THF (40 mL, 0.05 M) under Ar atmosphere at −20 °C. Triethylamine (1.1 mL, 8.0 mmol, 4.0 eq.) and miristoyl chloride (1.7 mL, 6.4 mmol, 3.2 eq.) were added dropwise to the solution, then also 4-dimethylaminopyridine (24 mg, 0.2 mmol, 0.1 eq.) was added. The reaction was stirred over 2 h, then controlled by TLC (DCM/MeOH 95:5; Rf product: 0.98). Subsequently, the solution was diluted in EtOAc and washed with 1 M HCl. The organic phase thus obtained was dried with Na_2_SO_4_ and the solvent was removed by rotavapor. The crude product thus obtained (4 g) was purified by flash chromatography (Hep/EtOAc 93:7; Rf Product: 0.35). After purification, 1.80 g of compound **13a** was obtained, in 77% yield.

^1^H NMR (400 MHz, CDCl_3_) δ 5.72 (d, *J*_*H-1*, *H-2*_ *= 9*.*3 Hz*, 1H, H-1), 5.30 (m, 2H, NH, H-3), 5.08 (t, *J*_*H-4*, *H-3*_ *= 9*.*8 Hz*, 1H, H-4), 4.31 – 4.21 (m, 1H, H-2), 4.02 (dd, *J*_*H-5*, *H-4*_ *= 9*.*8*, *J*_*H-5*, *H-6a*_ *= 4*.*4 Hz*, 1H, H-5), 3.66 (d, *J*_*H-6*, *H-5*_ *= 3*.*8 Hz*, 2H, H-6), 2.28 – 2.20 (m, 4H; CH_2_α chains), 2.12 (m, 2H; CH_2_α chains), 1.56 (s, 14H; CH_2_β chains), 1.24 (d, J = 2.9 Hz, 74H; chains bulk), 0.94 – 0.76 (m, 21H; 12x CH_3_ chains + 9x tBu-Si), 0.04 (m, 6H; Me-Si).

^13^C NMR (101 MHz, CDCl_3_) δ 174.3, 173.0, 172.0, 91.7, 77.3, 77.0, 76.7, 70.7, 70.7, 68.3, 62.6, 52.2, 36.8, 34.2, 34.2, 31.9, 29.7, 29.7, 29.7, 29.6, 29.6, 29.5, 29.5, 29.5, 29.4, 29.3, 29.3, 29.2, 29.2, 25.9, 25.9, 25.6, 25.0, 24.9, 22.7, 18.4, 14.1, −5.3.

HRMS (ESI-Q-TOF): m/z [M + Na^+^] calculated for C_68_H_131_NNaO_9_Si^+^: 1156.9485. Found: 1156.9478.

$$\alpha_{D}=+32.2$$

### 4.6 Compound 13b

*1,3,4-tri-O-dodecanoyl-2-dodecanamido-2-deoxy-6-O-tert-butyldimethylsilyl-α-d-glucopyranose.*

Compound **12b** (2.0 g, 4.2 mmol, 1 eq.) was dissolved in anhydrous THF (84 mL, 0.05 M) under Ar atmosphere. Triethylamine (2.3 mL, 16.8 mmol, 4.0 eq.) and lauroyl chloride (3.2 mL, 13.4 mmol, 3.2 eq.) were added dropwise to the solution, then also 4-dimethylaminopyridine (1.63 g, 13.4 mmol, 3.2 eq.) was added. The reaction was stirred over 2 h, then controlled by TLC (DCM/MeOH 95:5; Rf product: 0.98). Subsequently, the solution was diluted in EtOAc and washed with 1 M HCl. The organic phase thus obtained was dried with Na_2_SO_4_ and the solvent was removed by rotavapor. The crude product thus obtained (4 g) was purified by flash chromatography (Hep/EtOAc 93:7; Rf Product: 0.31). After purification, 3.43 g of compound **13b** were obtained, in 82% yield.

^1^H NMR (400 MHz, CDCl_3_) δ 6.19 (d, *J*_*H-1*, *H-2*_ *= 3*.*7 Hz*, 1H; H-1), 5.51 (d, *J*_*NH*, *H-2*_ *= 8*.*8 Hz*, 1H; NH), 5.27 – 5.20 (m, 1H; H-3), 5.18 (m, 1H; H-4), 4.40 (ddd, *J*_*H-2*, *H-3*_ *= 10*.*6*, *J*_*H-2*, *NH*_ *= 8*.*9*, *J*_*H-2*, *H-1*_ *= 3*.*7 Hz*, 1H; H-2), 3.80 (ddd, *J*_*H-5*, *H-4*_ *= 9*.*6*, *J*_*H-5*, *H-6a*_ *= 4*.*5*, *J*_*H-5*, *H-6b*_ *= 2*.*7 Hz*, 1H; H-5), 3.68 – 3.61 (m, 2H; H-6), 2.38 (t, *J*_*CH2α*,_ _*CH2β*_ *= 7*.*5 Hz*, 2H; CH_2_α chain), 2.28 – 2.22 (m, 4H; CH_2_α chain), 2.07 (dt, *J*_*CH2α*,_ _*CH2β*_ *= 11*.*5*, *3*.*5 Hz*, 2H; CH_2_α chain), 1.70 – 1.60 (m, 3H; CH_2_β chains), 1.55 (ddd, *J*_*CH2β*, *CH2α*_ *= 10*.*9*, *9*.*6*, *3*.*1 Hz*, 7H; CH_2_β chains), 1.37 – 1.20 (m, 94H; chains bulk), 0.90 – 0.84 (m, 25H; 12x CH_3_ chains + 9x tBu-Si), 0.04 – −0.01 (m, 7H; Me-Si).

^13^C NMR (101 MHz, CDCl_3_) δ 174.8, 172.9, 171.7, 171.5, 90.5, 77.3, 77.0, 76.7, 72.7, 70.8, 67.6, 62.0, 51.3, 36.6, 34.2, 34.2, 34.1, 33.8, 31.9, 29.6, 29.6, 29.5, 29.4, 29.4, 29.3, 29.3, 29.2, 29.2, 29.1, 29.1, 29.1, 25.8, 25.5, 24.9, 24.9, 24.9, 24.7, 22.7, 18.2, 14.1, −5.4.

HRMS (ESI-Q-TOF): m/z [M + Na^+^] calculated for C_60_H_115_NNaO_9_Si^+^: 1044.8233. Found: 1044.8239.

$$\alpha_{D}=+10.5$$

### 4.7 Compound 14a

*2-tetradecanamido-2-deoxy-3,4-di-O-tetradecanoyl-6-O-tert-butyldimethylsilyl-β-d-glucopyranose.*

Compound **13a** (1.8 g, 1.6 mmol, 1 eq.) was dissolved in anhydrous THF (40 mL, 0.04 M) with 1% v/v of water. Acetic acid (460 μL, 8.0 mmol, 5.0 eq.) and ethylenediamine (1.6 mL, 24.0 mmol, 15.0 eq.) were added to the solution at 0 °C. The reaction was allowed to return to room temperature (20 °C) and stirred for 4 h, then controlled by TLC (Hep/EtOAc 9:1; Rf starting material: 0.00). Subsequently, the solution was diluted in EtOAc and washed three times with 1 M HCl and three times with NaHCO_3_. A white precipitate forms during the washings, which is removed by filtration and discarded (amide between lauric acid and ethylenediamine). The organic liquid phase thus obtained was dried with Na_2_SO_4_ and the solvent was removed by rotavapor. The crude product thus obtained (1.7 g) was purified by flash chromatography (Tol/EtOAc 85:15; Rf product: 0.21). After purification, 1.0 g of compound **14a** was obtained, in 68% yield.

^1^H NMR (400 MHz, CDCl_3_) δ 5.73 (d, *J*_*NH*, *H-2*_ *= 9*.*3 Hz*, 1H; NH), 5.34 – 5.22 (m, 2H; H-3 + H-1), 5.08 (m, 1H; H-4), 4.33 – 4.17 (m, 1H; H-2), 4.03 (ddd, *J*_*H-5*, *H-4*_ *= 10*.*1*, *J*_*H-5*, *H-6a*_ *= 4*.*5*, *J*_*H-5*, *H-6b*_ *= 3*.*2 Hz*, 1H; H-5), 3.69 – 3.62 (m, 2H; H-6), 2.26 – 2.20 (m, 4H; CH_2_α chain), 2.12 (td, *J*_*CH2α*,_ _*CH2β*_ *= 7*.*5*, *5*.*5 Hz*, 2H; CH_2_α chain), 1.64 – 1.50 (m, 7H; CH_2_β chains), 1.37 – 1.19 (m, 74H; chains bulk), 0.97 – 0.82 (m, 22H; 9x CH_3_ chains + 9x tBu-Si), 0.05 (t, *J = 3*.*9 Hz*, 7H; Me-Si).

^13^C NMR (101 MHz, CDCl_3_) δ 174.3, 173.0, 172.0, 91.7, 77.3, 77.0, 76.7, 70.8, 70.6, 68.4, 62.6, 52.2, 36.8, 34.2, 34.2, 34.1, 31.9, 29.7, 29.6, 29.6, 29.6, 29.5, 29.5, 29.4, 29.4, 29.3, 29.3, 29.3, 29.3, 29.2, 29.2, 25.9, 25.9, 25.6, 24.9, 24.9, 24.9, 22.7, 18.4, 14.1, −5.3, −5.4.

HRMS (ESI-Q-TOF): m/z [M^+^] calculated for C_54_H_105_NO_8_Si^+^: 946.7502. Found: 946.7494.

$$\alpha_{D}=+10.83$$

### 4.8 Compound 14b

*2-dodecanamido-2-deoxy-3,4-di-O-dodecanoyl-6-O-tert-butyldimethylsilyl-α-d-glucopyranose.*

Compound **13b** (1.5 g, 1.5 mmol, 1 eq.) was dissolved in anhydrous THF (38 mL, 0.04 M) with 1% v/v of water. Acetic acid (428 μL, 7.5 mmol, 5.0 eq.) and ethylenediamine (1.5 mL, 22.5 mmol, 15.0 eq.) were added to the solution at 0 °C. The reaction was allowed to return to room temperature (20 °C) and stirred for 4 h, then controlled by TLC (Hep/EtOAc 9:1; Rf starting material: 0.00). Subsequently, the solution was diluted in EtOAc and washed three times with 1 M HCl and three times with NaHCO_3_. A white precipitate forms during the washings, which is removed by filtration and discarded (amide between lauric acid and ethylenediamine). The organic liquid phase thus obtained was dried with Na_2_SO_4_ and the solvent was removed by rotavapor. The crude product thus obtained (1.4 g) was purified by flash chromatography (Tol/EtOAc 85:15; Rf product: 0.21). After purification, 882 mg of compound **14b** were obtained, in 72% yield.

^1^H NMR (400 MHz, CDCl_3_) δ 5.73 (d, *J*_*NH*, *H-2*_ *= 9*.*3 Hz*, 1H; NH), 5.34 – 5.22 (m, 2H; H-3 + H-1), 5.08 (m, 1H; H-4), 4.33 – 4.17 (m, 1H; H-2), 4.03 (ddd, *J*_*H-5*, *H-4*_ *= 10*.*1*, *J*_*H-5*, *H-6a*_ *= 4*.*5*, *J*_*H-5*, *H-6b*_ *= 3*.*2 Hz*, 1H; H-5), 3.69 – 3.62 (m, 2H; H-6), 2.26 – 2.20 (m, 4H; CH_2_α chain), 2.12 (td, *J*_*CH2α*,_ _*CH2β*_ *= 7*.*5*, *5*.*5 Hz*, 2H; CH_2_α chain), 1.64 – 1.50 (m, 7H; CH_2_β chains), 1.37 – 1.19 (m, 58H; chains bulk), 0.97 – 0.82 (m, 22H; 9x CH_3_ chains + 9x tBu-Si), 0.05 (t, *J = 3*.*9 Hz*, 7H; Me-Si).

^13^C NMR (101 MHz, CDCl_3_) δ 174.3, 173.0, 172.0, 91.7, 77.3, 77.0, 76.7, 70.8, 70.6, 68.4, 62.6, 52.2, 36.8, 34.2, 34.2, 34.1, 31.9, 29.7, 29.6, 29.6, 29.6, 29.5, 29.5, 29.4, 29.4, 29.3, 29.3, 29.3, 29.3, 29.2, 29.2, 25.9, 25.9, 25.6, 24.9, 24.9, 24.9, 22.7, 18.4, 14.1, −5.3, −5.4.

HRMS (ESI-Q-TOF): m/z [M^+^] calculated for C_48_H_93_NO_8_Si^+^: 839.6670. Found: 839.6667.

$$\alpha_{D}=-16.5$$

### 4.9 Compound 15a

*1-(dibenzyl)phosphor-2-tetradecanamido-2-deoxy-3,4-di-O-tetradecanoyl-6-O-tert-butyldimethylsilyl-α-d-glucopyranose.*

Compound **14a** (690 mg, 0.75 mmol, 1 eq.) and imidazole triflate (436 mg, 1.7 mmol, 2.25 eq.) were dissolved in DCM (37.5 mL, 0.02 M) under inert atmosphere. Dibenzyl N,N-diisopropylphosphoramidite (570 mg, 1.65 mmol, 2.2 eq) was added to the solution at 0 °C. The reaction was monitored by TLC (Hep/EtOAc 8:2, Rf product: 0.15); after 30 min, substrate depletion was detected. The solution was then cooled at −20 °C and a solution of meta-chloroperbenzoic acid (516 mg, 3.0 mmol, 4 eq.) in 5 mL of DCM was added dropwise. After 30 min the reaction was allowed to return to RT (20 °C) and left stirring overnight. After TLC analysis (Hep/EtOAc 8:2; Rf product: 0.29), the reaction was quenched with 15 mL of a saturated NaHCO_3_ solution and concentrated by rotavapor. The mixture was then diluted in EtOAc and washed 3 times with a saturated NaHCO_3_ solution and three times with a 1 M HCl solution. The organic phase was recovered, dried with Na_2_SO_4_, and the solvent was removed by rotavapor. The crude thus obtained was purified by flash column chromatography (Hep/EtOAc 8:2; Rf product: 0.29). In total, 810 mg of pure compound **15a** were obtained as a yellow oil in a 91% yield.

^1^H NMR (400 MHz, CDCl_3_) δ 7.42 – 7.29 (m, 11H; aromatics), 5.70 (dd, *J*_*H-1*, *P-1*_ *= 6*.*0*, *J*_*H-1*, *H*,*2*_ *= 3*.*2 Hz*, 1H; H-1), 5.62 (d, *J*_*NH*, *H-2*_ *= 9*.*1 Hz*, 1H; N-H), 5.25 – 5.15 (m, 2H; H-4 + H-3), 5.13 – 4.95 (m, 4H; CH_2_Ph), 4.33 (ddt, *J*_*H-2*, *H-3*_ *= 10*.*6*, *J*_*H-2*, *NH*_ *= 9*.*0*, *J*_*H-2*, *H-1*_ *= 3*.*2 Hz*, 1H; H-2), 3.90 (dt, *J*_*H-5*, *H-4*_ *= 8*.*2*, *J*_*H-5*, *H-6*_ *= 3*.*8 Hz*, 1H; H-5), 3.61 – 3.51 (m, 2H; H-6), 2.28 – 2.17 (m, 4H; CH_2_α chain), 1.85 (hept, *J*_*CH2α*,_ _*CH2β*_ *= 7*.*48*, *7*.*48*, *7*.*48*, *7*.*48*, *7*.*31*, *7*.*31 Hz*, 2H; CH_2_α chain), 1.61 – 1.47 (m, 5H; CH_2_β chains), 1.47 – 1.37 (m, 2H; CH_2_β chains), 1.33 – 1.10 (m, 70H; chains bulk), 0.91 – 0.82 (m, 18, 9x CH_3_ chains + 9x tBu-Si), −0.02 (d, *J = 5*.*9 Hz*, 6H; Me-Si).

^13^C NMR (101 MHz, CDCl_3_) δ 174.2, 173.1, 171.6, 135.5, 135.4, 135.4, 135.3, 128.8, 128.7, 128.7, 128.4, 128.2, 128.0, 127.8, 96.8, 96.7, 77.3, 77.0, 76.7, 72.4, 70.2, 69.8, 69.7, 69.7, 69.7, 67.1, 61.4, 51.9, 51.8, 36.3, 34.2, 34.1, 31.9, 29.7, 29.7, 29.7, 29.6, 29.5, 29.5, 29.4, 29.3, 29.2, 29.2, 29.1, 25.8, 25.8, 25.4, 24.9, 24.9, 22.7, 18.3, 14.1, −5.5, −5.5.

^31^P NMR (162 MHz, CDCl_3_) δ −2.49 (s, 1P, P-1).

HRMS (ESI-Q-TOF): m/z [M + Na^+^] calculated for C_68_H_118_NNaO_11_PSi^+^: 1206.8104. Found: 1206.8113.

$$\alpha_{D}=+3.0$$

### 4.10 Compound 15b

*1-(dibenzyl)phosphor-2-dodecanamido-2-deoxy-3,4-di-O-dodecanoyl-6-O-tert-butyldimethylsilyl-α-d-glucopyranose.*

Compound **14b** (2.12 g, 2.4 mmol, 1 eq.) and imidazole triflate (1.4 g, 5.4 mmol, 2.25 eq.) were dissolved in DCM (121 mL, 0.02 M) under inert atmosphere. Dibenzyl N,N-diisopropylphosphoramidite (1.83 g, 5.3 mmol, 2.2 eq) was added to the solution at 0 °C. The reaction was monitored by TLC (Hep/EtOAc 8:2); after 30 min, substrate depletion was detected. The solution was then cooled at −20 °C and a solution of meta-chloroperbenzoic acid (1.66 g, 9.7 mmol, 4 eq.) in 17 mL of DCM was added dropwise. After 30 min the reaction was allowed to return to RT (20 °C) and left stirring overnight. After TLC analysis, the reaction was quenched with 15 mL of a saturated NaHCO_3_ solution and concentrated by rotavapor. The mixture was then diluted in EtOAc and washed 3 times with a saturated NaHCO_3_ solution and three times with a 1 M HCl solution. The organic phase was recovered, dried with Na_2_SO_4_, and the solvent was removed by rotavapor. The crude thus obtained was purified by flash column chromatography (Hep/EtOAc 8:2; Rf product: 0.29). In total, 2.41 g of pure compound **15a** was obtained as a yellow oil in a 91% yield.

^1^H NMR (400 MHz, CDCl_3_) δ 7.45 – 7.29 (m, 10H; aromatics), 5.70 (dd, *J*_*H-1*, *P-1*_ *= 5*.*9*, *J*_*H-1*, *H*,*2*_ *= 3*.*2 Hz*, 1H; H-1), 5.60 (d, *J*_*NH*, *H-2*_ *= 9*.*1 Hz*, 1H; N-H), 5.25 – 5.13 (m, 2H; H-4 + H-3), 5.13 – 4.97 (m, 4H; CH_2_Ph), 4.33 (ddt, *J*_*H-2*, *H-3*_ *= 10*.*6*, *J*_*H-2*, *NH*_ *= 9*.*0*, *J*_*H-2*, *H-1*_ *= 3*.*1 Hz*, 1H; H-2), 3.90 (dt, *J*_*H-5*, *H-4*_ *= 10*.*8*, *J*_*H-5*, *H-6*_ *= 3*.*1 Hz*, 1H; H-5), 3.56 (dd, *J*_*H-6*, *H-5*_ *= 3*.*1*, ^*2*^ *J = 1*.*8 Hz*, 2H; H-6), 2.28 – 2.17 (m, 4H; CH_2_α chain), 1.85 (hept, *J*_*CH2α*,_ _*CH2β*_ *= 7*.*48*, *7*.*48*, *7*.*48*, *7*.*48*, *7*.*31*, *7*.*31 Hz*, 2H; CH_2_α chain), 1.61 – 1.46 (m, 5H; CH_2_β chains), 1.46 – 1.36 (m, 2H; CH_2_β chains), 1.33 – 1.10 (m, 50H; chains bulk), 0.92 – 0.82 (m, 18, 9x CH_3_ chains + 9x tBu-Si), −0.02 (d, *J = 5*.*9 Hz*, 6H; Me-Si).

^13^C NMR (101 MHz, CDCl_3_) δ 174.2, 173.1, 171.6, 135.5, 135.4, 135.3, 129.0, 128.9, 128.8, 128.7, 128.7, 128.2, 128.0, 125.3, 96.8, 96.7, 77.3, 77.0, 76.7, 72.4, 70.2, 69.8, 69.7, 69.7, 69.6, 67.1, 61.5, 51.9, 51.8, 36.3, 34.2, 34.1, 31.9, 29.6, 29.6, 29.6, 29.5, 29.4, 29.3, 29.3, 29.3, 29.2, 29.2, 29.1, 25.8, 25.4, 24.9, 24.9, 22.7, 18.3, 14.1, −5.5, −5.5.

^31^P NMR (162 MHz, CDCl_3_) δ −2.51 (s, 1P, P-1).

HRMS (ESI-Q-TOF): m/z [M + Na^+^] calculated for C_62_H_106_NNaO_11_PSi^+^: 1122.7165. Found: 1122.7152.

$$\alpha_{D}=+29.8$$

### 4.11 Compound 16a

*1-(dibenzyl)phosphor-2-tetradecanamido-2-deoxy-3,4-di-O-tetradecanoyl-α-d-glucopyranose.*

Compound **15a** (500 mg, 0.42 mmol, 1 eq.) was dissolved in acetone (8.4 mL, 0.05 M) and IRC 120 H^+^ (3.75 g, 750% m/m) was added at RT (20 °C). The solution was left stirring for 48 h and monitored by TLC (Hep/Acetone 8:2; Rf product: 0.35). After reaction completion, the solution was filtered to remove the resin. The organic phase thus obtained was evaporated by rotavapor. The crude product thus obtained was purified by flash column chromatography (Hep/Acetone 85:15; Rf Product: 0.31). After purification, 247 mg of compound **16b** was obtained as a white solid in a 55% yield.

^1^H NMR (400 MHz, CDCl_3_) δ 7.42 – 7.29 (m, 9H; aromatics), 5.70 (dd, *J*_*H-1*, *P-1*_ *= 5*.*6*, *J*_*H-1*, *H-2*_ *= 3*.*3 Hz*, 1H; H-1), 5.59 (d, *J*_*NH*, *H-2*_ *= 9*.*1 Hz*, 1H; NH), 5.24 (dd, *J*_*H-3*, *H-2*_ *= 10*.*9*, *J*_*H-3*, *H-4*_ *= 9*.*6 Hz* 1H; H-3), 5.14 – 4.99 (m, 5H; CH_2_Ph + H-4), 4.40 – 4.30 (m, 1H; H-2), 3.81 (dt, *J*_*H-5*, *H-4*_ *= 10*.*3 Hz*, *J*_*H-5*, *H-6a*_ *= 4*.*1*, *J*_*H-5*, *H-6b*_ *= 2*.*2*, 1H; H-5), 3.53 (dd, *J*_*H-6a*, *H-6b*_ *= 13*.*0*, *J*_*H-6a*, *H-5*_ *= 2*.*2 Hz*, 1H; H-6b), 3.44 (dd, *J*_*H-6b*, *H-6a*_ *= 13*.*0*, *J*_*H-6b*, *H-5*_ *= 4*.*1 Hz*, 1H; H-6a), 2.33 – 2.19 (m, 4H; CH_2_α chain), 1.98 – 1.81 (m, 2H; CH_2_α chain), 1.62 – 1.38 (m, 7H; CH_2_β chains), 1.47 – 1.39 (m, 2H; CH_2_β chains), 1.32 – 1.17 (m, 61H; chains bulk), 0.92 – 0.84 (m, 9H; CH_3_ chains).

^13^C NMR (101 MHz, CDCl_3_) δ 128.5, 128.4, 127.8, 77.3, 77.0, 76.7, 69.2, 69.1, 48.9, 36.7, 34.2, 34.2, 31.9, 29.7, 29.6, 29.5, 29.4, 29.3, 29.2, 29.1, 25.6, 24.9, 24.7, 22.7, 14.1.

^31^P NMR (162 MHz, CDCl_3_) δ −2.32 (s, 1P, P-1).

HRMS (ESI-Q-TOF): m/z [M + Na^+^] calculated for C_62_H_104_NNaO_11_P^+^: 1092.7239. Found: 1092.7228.

$$\alpha_{D}=+40.2$$

### 4.12 Compound 16b

*1-(dibenzyl)phosphor-2-dodecanamido-2-deoxy-3,4-di-O-dodecanoyl-α-d-glucopyranose.*

Compound **15a** (500 mg, 0.45 mmol, 1 eq.) was dissolved in acetone (9.0 mL, 0.05 M) and IRC 120 H^+^ (3.75 g, 750% m/m) was added at RT (20 °C). Solution was left stirring for 48 h and monitored by TLC (Hep/Acetone 8:2; Rf product: 0.35). After reaction completion, the solution was filtered to remove the resin. Organic phase thus obtained was evaporated by rotavapor. Crude product thus obtained was purified by flash column chromatography (Hep/Acetone 85:15; Rf Product: 0.31). After purification, 244 mg of compound **16b** was obtained as a white solid in a 55% yield.

^1^H NMR (400 MHz, CDCl_3_) δ 7.42 – 7.29 (m, 10H; aromatics), 5.70 (dd, *J*_*H-1*, *P-1*_ *= 5*.*4*, *J*_*H-1*, *H-2*_ *= 3*.*4 Hz*, 1H; H-1), 5.61 (d, *J*_*NH*, *H-2*_ *= 9*.*0 Hz*, 1H; NH), 5.27 – 5.20 (dd, *J*_*H-3*, *H-2*_ *= 10*.*9*, *J*_*H-3*, *H-4*_ *= 9*.*5 Hz* 1H; H-3), 5.13 – 5.00 (m, 5H; benzylics + H-4), 4.39 – 4.31 (m, 1H; H-2), 3.81 (dt, *J*_*H-5*, *H-4*_ *= 10*.*2 Hz*, 1H; H-5), 3.53 (dd, *J*_*H-6a*, *H-6b*_ *= 12*.*9*, *J*_*H-6a*, *H-5*_ *= 1*.*7 Hz*, 1H; H-6a), 3.44 (dd, *J*_*H-6b*, *H-6a*_ *= 13*.*0*, *J*_*H-6b*, *H-5*_ *= 4*.*0 Hz*, 1H; H-6b), 2.26 (ddd, *J*_*CH2α*,_ _*CH2β*_ *= 17*.*3*, *11*.*4*, *4*.*6 Hz*, 5H; CH_2_α chain), 1.97 – 1.81 (m, 2H; CH_2_α chain), 1.62 – 1.49 (m, 5H; CH_2_β chains), 1.47 – 1.39 (m, 2H; CH_2_β chains), 1.32 – 1.17 (m, 55H; chains bulk), 0.88 (t, *J = 6*.*7 Hz*, 10H; CH_3_ chains).

^13^C NMR (101 MHz, CDCl_3_) δ 174.0, 173.1, 135.4, 135.3, 135.2, 128.9, 128.8, 128.7, 128.1, 128.0, 96.5, 96.4, 77.3, 77.0, 76.7, 72.0, 70.0, 69.9, 69.9, 69.8, 69.5, 67.6, 60.7, 51.9, 51.8, 40.8, 36.3, 34.1, 34.1, 31.9, 29.7, 29.6, 29.6, 29.6, 29.5, 29.4, 29.3, 29.3, 29.2, 29.1, 29.1, 28.4, 25.4, 24.9, 23.8, 22.7, 20.8, 17.5, 17.3, 14.6, 14.1.

^31^P NMR (162 MHz, CDCl_3_) δ −2.32 (s, 1P, P-1).

HRMS (ESI-Q-TOF): m/z [M + Na^+^] calculated for C_56_H_92_NNaO_11_P^+^: 1008.6300. Found: 1008.6306.

$$\alpha_{D}=+23.2$$

### 4.13 Compound FP11

*1-phospho-2-tetradecanamido-2-deoxy-3,4-di-O-tetradecanoyl-α-d-glucopyranose (sodium salt).*

Compound **16** (50 mg, 0.05 mmol, 1 eq.) dissolved in a 1:1 mixture of MeOH and DCM (5 mL, 0.01 M) was put under inert atmosphere. Palladium on carbon (10 mg, 20% m/m) was added to the solution. The reaction environment was put under vacuum, then H_2_ atmosphere was added. The solution was stirred for 2 h, H_2_ was removed and the reaction was monitored by TLC (EtPet/acetone 8:2; Rf product: 0.00). TEA (100 μL, 2% v/v) was added to the mixture, and the reaction was stirred for 15 min. The solution was filtered on syringe filters PALL 4549T Acrodisc 25 mm with GF/0.45 µm Nylon to remove the catalyst and solvents were evaporated by rotavapor. The crude was resuspended in a 1:1 DCM/MeOH solution and IRC 120 H^+^ was added. After 30 min stirring, IRC 120 H^+^ was removed by filtration, and the solution was evaporated. The crude was again dissolved in 1:1 DCM/MeOH solution and IRC 120 Na^+^ was added. After 30 min stirring, IRC 120 Na^+^ was filtered and solvents were removed by rotavapor. The crude product was purified through reverse chromatography employing a C4 functionalized column (PUREZZA-Sphera Plus Standard Flash Cartridge C4 - 25um - Size 25 g) in the Biotage^®^ Isolera LS System (gradient: H_2_O/THF 70:30 to 15:85 over 10 CV with 1% of an aqueous solution of Et_3_NHCO_3_ at pH 7.5; Retention time: 12–14 min). In total, 45 mg of **FP11** was obtained as a white powder in a quantitative yield.

^1^H NMR (400 MHz, MeOD) δ 5.58 (dd, *J*_*H-1*, *P-1*_ *= 6*.*6*, *J*_*H-1*, *H-2*_ *= 3*.*4 Hz*, 1H; H-1), 5.34 (dd, *J*_*H-3*, *H-2*_ *= 10*.*9 J*_*H-3*, *H-4*_ *= 9*.*4 Hz*, 1H; H-3), 5.15 (dd, *J*_*H-4*, *H-5*_ *= 10*.*3 J*_*H-4*, *H-3*_ *= 9*.*4 Hz*, 1H; H-4), 4.35 (dt, *J*_*H-2*, *H-3*_ *= 10*.*9*, *J*_*H-2*, *H-1*_ *= 3*.*2 Hz*, 1H; H-2), 4.08 (ddd, *J*_*H-5*, *H-4*_ *= 10*.*3*, *J*_*H-5*, *H-6b*_ *= 4*.*7*, *J*_*H-5*, *H-6a*_ *= 2*.*4 Hz*, 1H; H-5), 3.68 (dd, *J*_*H-6a*, *H-6b*_ *= 12*.*4*, *J*_*H-6a*, *H-6b*_ *= 2*.*3 Hz*, 1H; H-6a), 3.57 (dd, *J*_*H-6b*, *H-6a*_ *= 12*.*3*, *J*_*H-6b*, *H-5*_ *= 4*.*7 Hz*, 1H; H-6b), 2.40 – 2.15 (m, 6H; CH_2_α chain), 1.65 – 1.51 (m, 6H; CH_2_β chains), 1.30 (s, 64H; chains bulk), 0.94 – 0.87 (m, 9H; CH_3_ chains).

^13^C NMR (101 MHz, MeOD) δ 175.1, 173.1, 172.5, 94.6, 71.2, 70.6, 68.5, 60.3, 51.7, 51.6, 48.2, 48.0, 47.8, 47.6, 47.4, 47.2, 47.0, 35.6, 33.7, 33.6, 31.7, 31.7, 29.4, 29.4, 29.4, 29.3, 29.3, 29.2, 29.2, 29.2, 29.1, 29.1, 29.1, 29.0, 29.0, 28.9, 28.8, 25.6, 25.5, 24.5, 24.5, 22.3, 13.0.

^31^P NMR (162 MHz, MeOD) δ −1.93 (s, 1P, P-1).

HRMS ESI-MS: [M-H]^-^ calculated for C_48_H_91_NO_11_P^−^ m/z = 888.6335; found: m/z = 888.6328.

$$\alpha_{D}=+56.5$$

### 4.14 Compound FP18

*1-phospho-2-dodecanamido-2-deoxy-3,4-di-O-dodecanoyl-α-d-glucopyranose (sodium salt).*

Compound **16** (50 mg, 0.05 mmol, 1 eq.) dissolved in a 1:1 mixture of MeOH and DCM (5 mL, 0.01 M) was put under inert atmosphere. Palladium on carbon (10 mg, 20% m/m) was added to the solution. The reaction environment was put under vacuum, then H_2_ atmosphere was added. The solution was stirred for 2 h, H_2_ was removed and the reaction was monitored by TLC (EtPet/acetone 8:2; Rf product: 0.00). TEA (100 μL, 2% v/v) was added to the mixture, and the reaction was stirred for 15 min. The solution was filtered on syringe filters PALL 4549T Acrodisc 25 mm with GF/0.45 µm Nylon to remove the catalyst and solvents were evaporated by rotavapor. The crude was resuspended in a 1:1 DCM/MeOH solution and IRC 120 H^+^ was added. After 30 min stirring, IRC 120 H^+^ was removed by filtration and the solution was evaporated. The crude was again dissolved in 1:1 DCM/MeOH solution and IRC 120 Na^+^ was added. After 30 min stirring, IRC 120 Na^+^ was filtered and solvents were removed by rotavapor. The crude product was purified through reverse chromatography employing a C4 functionalized column (PUREZZA-Sphera Plus Standard Flash Cartridge C4 - 25um - Size 25 g) in the Biotage^®^ Isolera LS System (gradient: H_2_O/THF 70:30 to 15:85 over 10 CV with 1% of an aqueous solution of Et_3_NHCO_3_ at pH 7.5; Retention time: 12–14 min). In total, 45 mg of **FP18** was obtained as a white powder in a quantitative yield.

^1^H NMR (400 MHz, MeOD) δ 5.57 (dd, *J*_*H-1*, *P-1*_ *= 6*.*6*, *J*_*H-1*, *H-2*_ *= 3*.*4 Hz*, 1H; H-1), 5.35 (dd, *J*_*H-3*, *H-2*_ *= 10*.*8*, *J*_*H-3*, *H-4*_ *= 9*.*3 Hz*, 1H; H-3), 5.15 (dd, *J*_*H-4*, *H-5*_ *= 10*.*1 J*_*H-4*, *H-3*_ *= 9*.*5 Hz*, 1H; H-4), 4.34 (dt, *J*_*H-2*, *H-3*_ *= 10*.*9*, *J*_*H-2*, *H-1*_ *= 2*.*9 Hz*, 1H; H-2), 4.10 (ddd, *J*_*H-5*, *H-4*_ *= 10*.*4*, *J*_*H-5*, *H-6b*_ *= 4*.*6*, *J*_*H-5*, *H-6a*_ *= 2*.*3 Hz*, 1H; H-5), 3.68 (dd, *J*_*H-6a*, *H-6b*_ *= 12*.*5*, *J*_*H-6a*, *H-6b*_ *= 2*.*4 Hz*, 1H; H-6a), 3.57 (dd, *J*_*H-6b*, *H-6a*_ *= 12*.*3*, *J*_*H-6b*, *H-5*_ *= 4*.*7 Hz*, 1H; H-6b), 2.40 – 2.12 (m, 7H; CH_2_α chain), 1.65 – 1.51 (m, 7H; CH_2_β chains), 1.32 (s, 55H; chains bulk), 1.00 – 0.82 (m, 10H; CH_3_ chains).

^13^C NMR (101 MHz, MeOD) δ 175.1, 173.1, 172.5, 94.6, 71.2, 70.6, 68.5, 60.3, 51.7, 51.6, 48.2, 48.0, 47.8, 47.6, 47.4, 47.2, 47.0, 35.6, 33.7, 33.6, 31.7, 31.7, 29.4, 29.4, 29.4, 29.3, 29.3, 29.2, 29.2, 29.2, 29.1, 29.1, 29.1, 29.0, 29.0, 28.9, 28.8, 25.6, 25.5, 24.5, 24.5, 22.3, 13.0.

^31^P NMR (162 MHz, MeOD) δ −1.88 (s, 1P, P-1).

HRMS ESI-MS: [M-H]^-^ calculated for C_42_H_79_NO_11_P^−^ m/z = 804.5396; found: m/z = 804.5401.

$$\alpha_{D}=+24.2$$

## Data Availability

The datasets presented in this study can be found in online repositories. The names of the repository/repositories and accession number(s) can be found in the article/Supplementary Material.
