# Supplementary material for: Cleaner synthesis of preclinically validated vaccine adjuvants
Source: Front Chem. 2023 Nov 2;11:1252996. doi: 10.3389/fchem.2023.1252996 (PMC10651716; doi:10.3389/fchem.2023.1252996)
Supplement: Supplementary file 1 [file DataSheet1.docx]

Supplementary Material

Cleaner synthesis of preclinically validated vaccine adjuvants

**Index**

The datasets presented in this study can be found in Bicocca Open Archive Research Data.

[1 NMR Spectra 2](#_Toc145689375)

[1.1 Compound 11a (^1^H, ^13^C) 2](#_Toc145689376)

[1.2 Compound 12a (^1^H, ^13^C) 3](#_Toc145689377)

[1.3 Compound 13a (^1^H, ^13^C) 4](#_Toc145689378)

[1.4 Compound 14a (^1^H, ^13^C) 5](#_Toc145689379)

[1.5 Compound 15a (^1^H, ^13^C, ^31^P) 6](#_Toc145689380)

[1.6 Compound 16a (^1^H, ^13^C, ^31^P) 8](#_Toc145689381)

[1.7 Compound FP11 (^1^H, ^13^C, ^31^P) 10](#_Toc145689382)

[1.8 Compound 11b (^1^H, ^13^C) 12](#_Toc145689383)

[1.9 Compound 12b (^1^H, ^13^C) 13](#_Toc145689384)

[1.10 Compound 13b (^1^H, ^13^C) 14](#_Toc145689385)

[1.11 Compound 14b (^1^H, ^13^C) 15](#_Toc145689386)

[1.12 Compound 15b (^1^H, ^13^C, ^31^P) 16](#_Toc145689387)

[1.13 Compound 16b (^1^H, ^13^C, ^31^P) 18](#_Toc145689388)

[1.14 Compound FP18 (^1^H, ^13^C, ^31^P) 20](#_Toc145689389)

# NMR Spectra

## Compound 11a (^1^H, ^13^C)

## Compound 12a (^1^H, ^13^C)

## Compound 13a (^1^H, ^13^C)

## Compound 14a (^1^H, ^13^C)

## Compound 15a (^1^H, ^13^C, ^31^P)

## Compound 16a (^1^H, ^13^C, ^31^P)


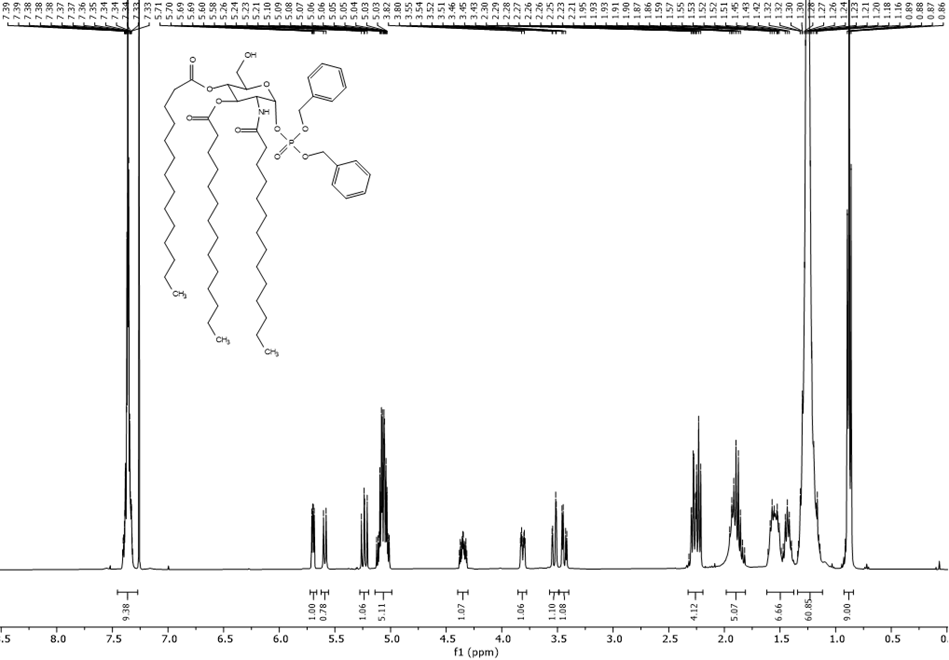

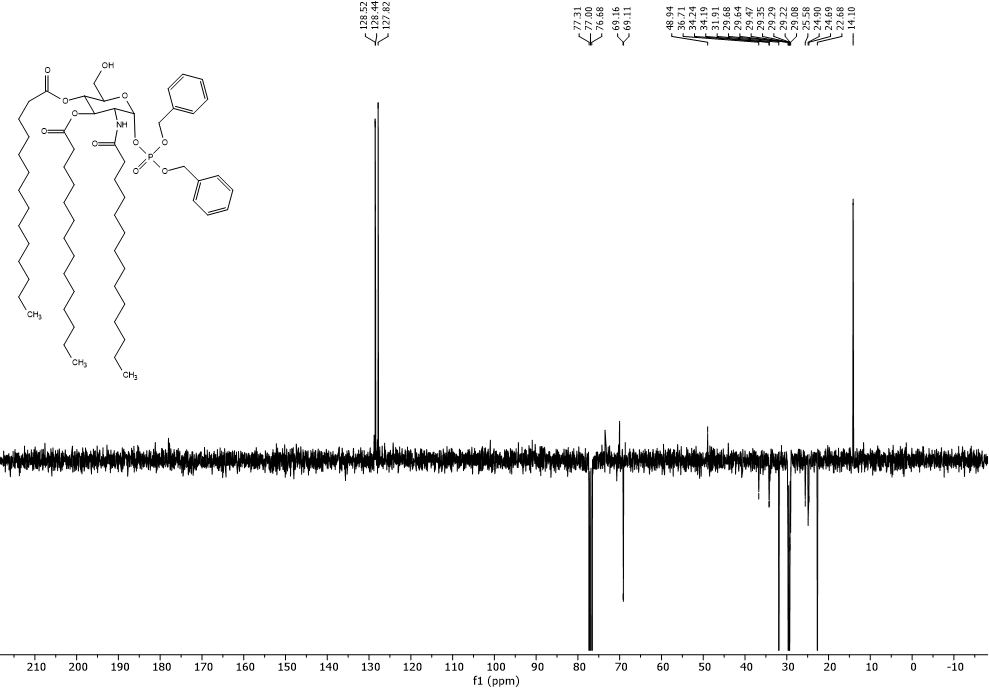

## Compound FP11 (^1^H, ^13^C, ^31^P)


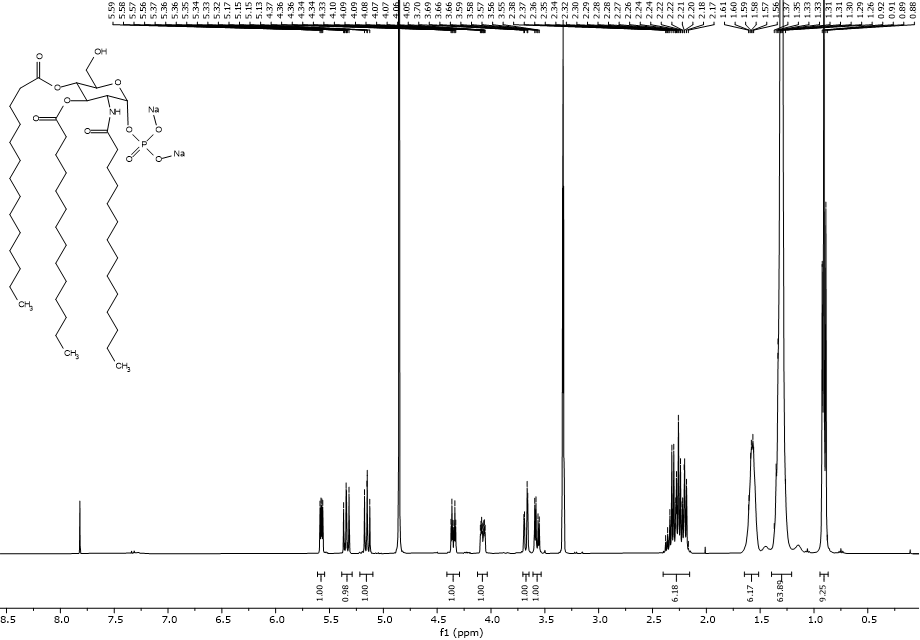

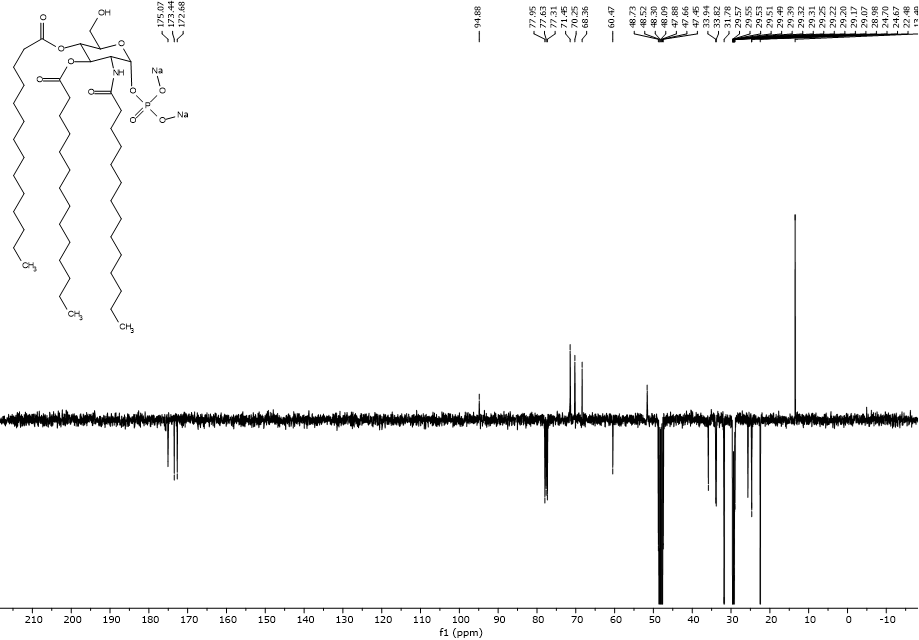

## Compound 11b (^1^H, ^13^C)

## Compound 12b (^1^H, ^13^C)

## Compound 13b (^1^H, ^13^C)

## Compound 14b (^1^H, ^13^C)

## Compound 15b (^1^H, ^13^C, ^31^P)

## Compound 16b (^1^H, ^13^C, ^31^P)

## Compound FP18 (^1^H, ^13^C, ^31^P)
